# Ghrelin, Neuroinflammation, Oxidative Stress, and Mood Disorders: What Are the Connections?

**DOI:** 10.2174/1570159X22999240722095039

**Published:** 2024-07-22

**Authors:** Jessica Mingardi, Ramona Meanti, Caterina Paoli, Carlo Cifani, Antonio Torsello, Maurizio Popoli, Laura Musazzi

**Affiliations:** 1 School of Medicine and Surgery, University of Milano-Bicocca, Monza, Italy;; 2 Pharmacology Unit, School of Pharmacy, University of Camerino, Camerino, Italy;; 3 Department of Pharmaceutical Sciences, University of Milan, 20133 Milano, Italy

**Keywords:** Ghrelin, inflammation, stress, oxidative stress, antidepressant, gut-brain axis, mood disorders, GHS-R1a

## Abstract

Ghrelin is a gut peptide hormone associated with feeding behavior and energy homeostasis. Acylated ghrelin binds to the growth hormone secretagogue receptor 1a subtype (GHS-R1a) in the hippocampus, leading to GH release from the anterior pituitary. However, in recent years, ghrelin and its receptor have also been implicated in other processes, including the regulation of cardiomyocyte function, muscle trophism, and bone metabolism. Moreover, GHS-R1a is distributed throughout the brain and is expressed in brain areas that regulate the stress response and emotional behavior. Consistently, a growing body of evidence supports the role of ghrelin in regulating stress response and mood. Stress has consistently been shown to increase ghrelin levels, and despite some inconsistencies, both human and rodent studies suggested antidepressant effects of ghrelin. Nevertheless, the precise mechanism by which ghrelin influences stress response and mood remains largely unknown. Intriguingly, ghrelin and GHS-R1a were consistently reported to exert anti-inflammatory, antioxidant, and neurotrophic effects both *in vivo* and *in vitro*, although this has never been directly assessed in relation to psychopathology. In the present review we will discuss available literature linking ghrelin with the stress response and depressive-like behavior in animal models as well as evidence describing the interplay between ghrelin and neuroinflammation/oxidative stress. Although further studies are required to understand the mechanisms involved in the action of ghrelin on mood, we hypothesize that the anti-inflammatory and anti-oxidative properties of ghrelin may give a key contribution.

## 1 INTRODUCTION

Mood disorders, including Major Depressive Disorder (MDD), are the most common mental diseases worldwide [1]. Available antidepressants, classically acting by increasing monoamine bioavailability, retain significant limitations, including a delay of several weeks in the onset of therapeutic effect and a high percentage of treatment-resistant patients [2]. Our understanding of antidepressant pharmacotherapy has dramatically changed in recent years with the discovery that subanesthetic ketamine exerts antidepressant effects in a matter of minutes. This has paved the way for the development of rapid-acting antidepressants, including classic serotonergic psychedelics. Although the clinical benefits of ketamine and psychedelics are impressive, their potential for abuse limits their use [3, 4]. The study of etiopathogenetic mechanisms of depression is essential for the identification of new pharmacological targets that are required for the development of novel and safer therapeutic strategies. In this context, recent years have seen a growing interest in studying the gut-brain axis as a potential novel target for treating neuropsychiatric disorders [5, 6].

### 1.1 The Gut-brain Axis: Microbiota and Gut Peptides

The gut-brain axis is a bidirectional communication system enabling the gut to communicate with the brain and vice versa. The mechanisms mediating this communication are complex and not fully elucidated but include neural, endocrine, immune, and metabolic pathways (a detailed description is out of the scope of the present paper; for extensive reviews, please see [5-7]). In this context, increased emphasis has been given to the role of the microbiota and its metabolites in both health and disease. However, more recently, peptide hormones released from the gut, including glucagon-like peptide (GLP-1), peptide YY (PYY), cholecystokinin (CCK), corticotropin-releasing factor (CRF), oxytocin, and ghrelin [5], have also garnered attention. Indeed, the gastrointestinal tract is the largest endocrine organ in mammals, secreting dozens of different signaling molecules, including peptides. Importantly, gut peptide receptors are expressed not only in the hypothalamus, where they regulate appetite and food intake but also in cortico-limbic areas as well as on immune cells and vagus nerve terminals, thereby enabling indirect gut-brain communication. Moreover, the release of gut peptides can, in turn, be regulated by the gut microbiota, and the diversity and composition of the enteric bacteria can influence the release of gut peptides [5].

The present narrative review will focus on ghrelin, an orexigenic hormone that not only regulates food intake and energy balance but also has recognized roles in the control of different behaviors, including learning and memory, reward, and vulnerability to stress [8-11]. In detail, we will describe ghrelin's physiological functions, with a particular focus on possible implications in the pathophysiology of psychiatric disorders, including evidence regarding its functions in the regulation of depressive behavior. Although the link between ghrelin and depression has been clearly demonstrated at both preclinical and clinical levels (see section 3), the molecular mechanisms mediating its behavioral effects are still largely unknown. Here, we put forward the hypothesis that a major role could be played by the regulation of neuroinflammation and oxidative processes. Indeed, compelling evidence (reviewed in section 4) demonstrated that ghrelin not only has an anti-inflammatory effect mediated by a reduction of the secretion of inflammatory cytokines but also negatively regulates oxidative stress and mitochondrial dysfunction. Importantly, ghrelin exerts these functions both in peripheral tissues and in the central nervous system [12]. Given the recognized role of neuroinflammation and oxidative stress in the etiopathogenesis of mood disorders, we postulate that ghrelin actions at this level may give reason for its therapeutic potential in the management of these diseases. Data for this review were collected using the PubMed database.

## 2 GHRELIN SYNTHESIS AND REGULATION

Ghrelin is a 28 amino acid peptide hormone, synthesized by the X/A-like oxyntic gland cells in the gastric mucosa from its precursor pre-proghrelin, which is encoded by the GHRL gene, translated, and then cleaved into ghrelin. After its translation, ghrelin can be acylated by Ghrelin O-acyltransferase (GOAT), an enzyme expressed in the endoplasmic reticulum of ghrelin-producing cells. Both des-acyl-ghrelin and acyl-ghrelin are released in the bloodstream, and the latter is rapidly deacylated to des-acyl-ghrelin. Ghrelin is an orexigenic hormone and is mainly released in fasting conditions [8, 10, 13, 14]. The actions of acyl-ghrelin are mediated by binding to the growth hormone secretagogue receptor 1a (GHS-R1a), for which acyl-ghrelin is the only known ligand [15, 16]. GHS-R1a is a Gq-protein coupled receptor with strong, constitutive ligand-independent activity, while acyl-ghrelin further potentiates the receptor by stabilizing the active conformation of GHS-R1a. GHS-R1a is expressed both peripherally and in the central nervous system, where it is mostly detectable in the nuclei of the thalamus, the hippocampus, the midbrain, and the amygdala [8, 13, 14]. The GHRL gene also encodes for the GHS-R1b receptor, which contains only five transmembrane domains and whose function is still under investigation. Preliminary evidence shows that it may form heterodimers with GHS-R1a to act as negative feedback for acyl-ghrelin signaling [16, 17]. Importantly, GHS-R1a can also form heterodimers with other G-protein coupled receptors, including somatostatin 5, dopamine 1 and 3, serotonin 2C, and melanocortin 3 receptors, resulting in the activation of non-canonical signal transduction pathways. At the same time, several lines of evidence have revealed that the biological functions of des-acyl-ghrelin are mediated by a GHS-R1a-independent pathway, although the receptors for des-acyl-ghrelin remain unidentified [18].

### 2.1 Physiological Functions of Ghrelin

Although predominantly produced in the stomach, the majority of ghrelin functions are dependent on GHS-R1a located in the brain. The primary role of ghrelin is to regulate glucose homeostasis and stimulate food intake by activating hypothalamic neurons involved in homeostatic feeding [8, 13]. However, its functions go far beyond just regulating energy metabolism and food intake. Indeed, ghrelin signaling is also involved in the regulation of neuroendocrine and autonomic functions leading to behavioral changes. The effects of ghrelin are both direct (GHS-R1a-dependent) and indirect through the induction of growth hormone (GH) release in both the adenohypophysis and the hypothalamus. Importantly, a reciprocal relationship has been reported between ghrelin levels and the activation of the Hypothalamic-Pituitary-Adrenal (HPA) axis [19]. Indeed, ghrelin increases the release of corticotropin-releasing hormone (CRH) from the paraventricular nucleus, thus driving increased adrenocorticotropic hormone (ACTH) secretion from the pituitary and activating the stress response, while CRH decreases the expression of ghrelin [9, 19]. Ghrelin has been shown to increase vigilance and modulate mood, fear, and anxiety [10]. Indeed, ghrelin increases motivated behavior for food, and it has been hypothesized that when food is not readily available, it may decrease anxiety traits and not to hinder the animal from finding food [20]. Moreover, ghrelin has also recognized peripheral roles in the modulation of cardiac functions, muscle mass, bone metabolism, and cancer development and progression. For extensive reviews on ghrelin physiological functions, please see [8, 14, 21].

## 3 GHRELIN: LINK WITH STRESS AND MOOD DISORDERS

The impact of ghrelin on pathways orchestrating behaviors linked to reward, mood, anxiety, stress, and memory makes this hormone particularly interesting in the context of mood disorders. In this section, we will review the studies linking ghrelin to depression in both clinical and preclinical studies.

### 3.1 Ghrelin and Mood Disorders

The knowledge about ghrelin involvement in MDD comes from clinical studies that have shown alterations in the levels of this gut peptide in depressed patients and mood elevation following the administration of ghrelin either to depressed patients or healthy subjects [9-11].

Most of the studies reported higher levels of ghrelin in the plasma of MDD patients compared to healthy subjects [22-25], and this increase has been associated with the severity of depression, showing higher levels in patients with more severe symptomatology [24]. Interestingly, a sex effect was found in children with anxiety disorders, with higher plasma ghrelin levels in females compared to males [26].

Conversely, a few research groups reported no changes [27-29] or even decreased levels of both acyl- and des-acyl-ghrelin in depressed patients [30]. However, it is worth mentioning that not all the studies reported whether acyl-ghrelin, des-acyl-ghrelin, or total ghrelin were measured. Moreover, specific characteristics of the population examined, such as nutritional status, time of sample collection, age, sex, and drug treatments, might influence the results.

Further proof of the involvement of ghrelin in mood disorders is the fact that antidepressant drugs have been shown to modulate ghrelin expression, reducing its levels in the plasma of treated patients [22, 24, 25, 30, 31]. Notably, this effect was seen selectively in responder patients but not in non-responders, suggesting that ghrelin levels might be used as a predictor of treatment response [22, 25, 31]. Similarly, ghrelin serum levels were higher in non-responder subjects with panic disorder compared to responders and healthy controls [25].

The administration of ghrelin in healthy subjects, besides metabolic effects and hunger induction, was reported to elevate mood [32] and to increase plasma cortisol levels [33], while male but not female MDD patients who received acute ghrelin administration showed a trend for improvement of depressive symptoms including sleep disturbances [34].

Interestingly, allelic variants of the ghrelin gene were implicated in mechanisms of gene x environment interaction regulating the risk and symptom severity of psychiatric disorders. In an early study, the Leu72Met polymorphism in the pre-proghrelin gene was found to be less frequent in MDD patients than in healthy controls [35]. Moreover, male earthquake survivors carrying the Leu72Met polymorphism were reported to have a lower incidence of depression than male 72Leu/Leu homozygous, female 72Met allele carriers, or female 72Leu/Leu homozygotes [36]. At the same time, the Leu72Met polymorphism interacts with a polymorphism of the orexin gene to predict the risk of post-traumatic stress disorder (PTSD) in a sex-dependent manner [37]. For females, the lack of the pre-proghrelin 72Met allele is a risk factor for developing PTSD symptoms when the rs696217 genotype of the orexin gene is present; in contrast, the lack of the rs696217 of the orexin gene can be a risk factor for males only when the 72Met allele is present.

Since the Leu72Met polymorphism is located outside the coding region of mature ghrelin, it cannot change protein sequence but may instead influence mRNA stability or protein processing. However, the effects of the Leu72Met polymorphism on ghrelin functions are still poorly understood.

### 3.2 Ghrelin in Animal Models of Mood Disorders

#### 3.2.1 Ghrelin and Stress

A huge amount of literature has investigated the link between stress, ghrelin, and depressive-/anxious-like behaviors in animal models [10] (Table **1**). The first observation that suggested a role for ghrelin in behavioral outcomes goes back to 2001 when Asakawa and collaborators showed that ghrelin administration had an anxiogenic effect in mice that could be prevented using a CRH receptor antagonist [38], showing for the first time the interaction of ghrelin with the HPA axis. Moreover, they observed that acute stressors, such as tail pinch or fasting, increased ghrelin gene expression in the stomach [38].

Most of the following studies agreed to report increased levels of ghrelin in the plasma or serum of rodents exposed to different protocols of acute or chronic stress [39-54].

Only a few groups reported opposite results, showing a decrease in ghrelin expression in the serum of rats exposed to 5 weeks of chronic unpredictable mild stress (CUMS) [55] or both in the plasma of rats acutely injected with bacterial lipopolysaccharide [56] and mice exposed to chronic social defeat (CSDS) and Novelty stress [57, 58]. Of note, differences may depend on the specific form of ghrelin measured (acyl- *vs.* des-acyl-ghrelin, or total ghrelin), the species and strain used, as well as the feeding conditions and time of day in which the samples were collected.

Remarkably, also the expression of the ghrelin receptor GHS-R1a was consistently shown to increase under stress exposure, as demonstrated in the PVN of rats exposed to social stress in early life [59], in the hypothalamus of mice after chronic social stress [46], in the ventral hippocampus and prefrontal cortex of adult rats after postnatal stress [60], and in the hippocampus of both defeated and CUMS mice [40, 61]. Interestingly, one study suggested sex differences in the ghrelin axis with higher ghrelin and GHSR expression in the hippocampus and amygdala of female rats compared to males, which was associated with a stronger anxiolytic response to ghrelin [62].

To the best of our knowledge, only one study reported decreased levels of GHS-R1a mRNA and protein in the hippocampus and prefrontal cortex (despite increased blood levels of ghrelin) in female mice subjected to postpartum immobilization stress [48].

Interestingly, it has been proposed that the activation of ghrelin signaling in response to stress may be a homeostatic adaptation helping to cope with stress, but at the expense of increased caloric intake (Table **1**) [63-67].

#### 3.2.2 Antidepressant/Anxiolytic Properties of Ghrelin in Animal Models

Other groups studied the effect of exogenous ghrelin administration on depressive- and anxious-like behaviors in animal models (Table **2**).

Lutter and collaborators found that the subcutaneous administration of ghrelin, as well as increased endogenous ghrelin levels obtained by caloric restriction, significantly reduced anxious- and depressive-like behaviors induced by CSDS in C57BL/6J mice [49]. Similarly, the direct administration of ghrelin in the lateral ventricle of Albino Swiss mice that underwent olfactory bulbectomy induced a significant decrease in depressive-like behavior, as shown by a reduced immobility time in the tail suspension test [68]. Intracerebroventricular ghrelin administration was also shown to exert an anxiolytic effect in mice receiving tail pinch [38]. Two-week i.p. administration of acyl-ghrelin or intracerebroventricular injection of either ghrelin or of a GHS-R1a agonist improved depressive- and anhedonic-like behavior in mice and rats, respectively [61]. Repeated low-dose microinjections of ghrelin in the hippocampus were shown to alleviate depressive- and anxiety-like behaviors induced by CSDS in C57BL/6J mice [40], while the direct infusion in the dorsal hippocampus of male mice impaired memory acquisition [69]. Interestingly, Borchers and collaborators found sex differences in the effect of intraperitoneal acyl-ghrelin administration, with an anxiolytic effect being reported only in female rats [62]. Instead, the subcutaneous injection of a GHS-R1a antagonist in a mouse model of early-life stress limited the activation of the HPA axis induced by maternal separation in both male and female pups [41].

Interestingly, Gupta and collaborators showed that the subcutaneous injection of acyl-ghrelin or a ghrelin receptor agonist, both chronically during stress and acutely after stress, did not reverse social avoidance in C57BL/6N mice after ten days of CSDS [70].

#### 3.2.3 Genetic Manipulation of Ghrelin and Ghrelin Receptor

Another approach used to study the involvement of the ghrelin system in depressive-like behavior is the generation of transgenic lines in which the expression of ghrelin or GHS-R1a were manipulated (Table **3**) [39, 40, 49, 71-76]. Walker and collaborators demonstrated that GHSR-null mice (GHSR^-/-^) are more vulnerable to CSDS and develop a stronger depressive-like phenotype compared to controls [71]. Moreover, this behavior was paralleled by reduced cell proliferation and survival in the ventral dentate gyrus subgranular zone, suggesting the involvement of hippocampal neurogenesis in the effects of ghrelin on mood [71]. Similarly, GHSR -/- mice are more anxious after acute restraint stress, compared to wild-type [72] and the selective knockdown of GHSR in the hippocampus of male C57BL/6J mice exacerbated depressive- and anxious-like behaviors induced by CSDS [40].

Mice lacking both acyl- and des-acyl-ghrelin showed higher anxiety-like behaviors, whereas the anxious phenotype was attenuated in mice lacking acyl-ghrelin but expressing des-acyl-ghrelin. In the same study, the loss of GHS-R1a did not affect anxiety-like behavior, further suggesting that the effects of acyl- and des-acyl-ghrelin are not exclusively mediated by the canonical GHS-R1a signaling pathway [73]. However, in another study, both wild-type and GHSR^-/-^ mice showed a similar reduction in sociability after CSDS exposure, suggesting no effect of the genetic deletion on the anxious phenotype [74].

#### 3.2.4 Ghrelin and Antidepressant Treatments

A few antidepressant drugs have been reported to affect the ghrelin system. Treatment with Selective Serotonin Reuptake Inhibitors (SSRIs), including fluoxetine and paroxetine, decreased acyl-ghrelin levels in the plasma of rats through mechanisms involving the serotonin 2c receptor [77]. Moreover, both fluoxetine and clomipramine were shown to reduce short- and long-term memory retention promoted by ghrelin administration in rats [78]. The levels of serotonin appear to be critical in allowing or blunting the physiological activity of ghrelin in specific brain regions, including the hippocampus [78]. This interplay is supported by data showing ghrelin ability to inhibit depolarization-evoked serotonin release from hypothalamic synaptoneurosomes [79].

Notably, the ghrelin receptor seems to be required for the antidepressant activity of natural active compounds such as Meranzin hydrate and Paeoniflorin [80, 81]. Indeed, the deletion of GHS-R1a in rats prevented the ability of Meranzin hydrate to ameliorate depressive-like behaviors [81]. Similarly, mice lacking GHS-R1a and treated with Paeoniflorin showed a reduced rescue of depressive-like behaviors induced by CMS [80].

#### 3.2.5 The Paradox of Ghrelin

Taking together the clinical and preclinical evidence regarding the involvement of ghrelin in the modulation of mood, the picture appears somewhat paradoxical [9, 10]. Indeed, while most of the studies reported increased levels of active ghrelin in the plasma of patients with mood disorders and in stressed-based animal models of depression, exogenous ghrelin administration has been shown to exert antidepressant and anxiolytic effects. Moreover, the genetic silencing of ghrelin or GHS-R1a exacerbates the stress response in rodents exposed to different stress protocols and blunts the antidepressant effect of various treatments. Overall, experimental evidence suggests that ghrelin acts to modulate behavior and HPA axis function in a context-dependent fashion. One explanation might be that the increase of ghrelin following stress exposure could be a coping mechanism, but more investigations are required to better understand the association between ghrelin levels, GHS-R1a function, and depressive behavior.

## 4 GHRELIN, NEUROINFLAMMATION, AND OXIDATIVE STRESS

A wide body of literature has demonstrated the immunomodulatory and antioxidant properties of ghrelin [12].

The dynamic balance of pro- and anti-inflammatory signals has been reported to be crucial in controlling pathological risk and disease progression. Importantly, a persistent challenge to the immune system, referred to as low-grade neuroinflammation, is recognized as an etiological factor for a number of pathophysiologic processes and adverse health outcomes, including cardiovascular diseases, neurodegeneration, and mood disorders [82].

The brain possesses specialized immune cells called microglia that carry out macrophage-like functions and have the primary role of maintaining brain homeostasis and providing rapid responses to damage or infection. However, excess or prolonged inflammatory cytokine activity perturbs multiple neuronal functions, including neurotransmitter signaling and neuroplasticity processes, ultimately leading to structural, functional, and behavioral changes [83, 84]. Importantly, in conditions that weaken the blood-brain barrier, peripheral proinflammatory mediators can infiltrate the brain and contribute to the activation of microglial cells.

The redox balance is a finely tuned process that protects from excessive production of reactive oxygen species (ROS) and free radicals [85]. Conversely, the imbalance between ROS generation and the antioxidant system leads to oxidative stress and mitochondrial dysfunction, which, in turn, may induce neurotoxicity. The brain is vulnerable to oxidative stress because of its higher oxygen consumption, higher lipid content, and weaker antioxidative defense, and oxidative stress has been implicated in the pathogenesis of mood disorders [86].

Even though oxidative stress and neuroinflammation are two totally different pathological events, they are linked and affect one another [87]. Indeed, inflammatory cells secrete ROS, while some ROS can further promote intracellular signaling cascades, leading to increased expression of pro-inflammatory genes.

In the following paragraphs, we will describe the evidence showing the effects of ghrelin or ghrelin analogues on neuroinflammation and oxidative stress in the brain.

### 4.1 Ghrelin and Microglia Activation

Ghrelin has been extensively studied in the context of neuroinflammation using a wide range of different preclinical models [88], although models of mood disorders have never been considered. Particular attention has been given to its activity on microglia cells and on its putative anti-inflammatory properties [88, 89].

Indeed, treatment with ghrelin has been shown to exert anti-inflammatory effects *in vitro* and *in vivo* by blunting microglia activation and suppressing the p38 MAPK-JNK signaling pathway, resulting in a decreased production of inflammatory markers such as TNF-α, IL-6, and IL-1β [90, 91]. By preventing pro-inflammatory responses, ghrelin was reported to ameliorate cell survival in specific brain areas such as the hippocampus and the substantia nigra [92, 93]. Moreover, exogenous ghrelin has been associated with a reduced activation of NF-kB and nod-like receptor protein 3 (NLRP3) inflammasome signaling pathways [94].

Interestingly, contrasting results have been reported as to whether the effect of ghrelin is dependent or not on the activation of its receptor GHS-R1a [92, 93]. In fact, ghrelin anti-inflammatory properties have been observed in tissues expressing either GHS-R1a or GHS-R1b isoforms or neither of them, leading to the hypothesis of the existence of unknown ghrelin receptors or of heterodimeric forms with other G-protein coupled receptors, that may mediate its activity [95].

### 4.2 Ghrelin, Oxidative Stress, and Mitochondrial Dysfunctions

Ghrelin has been shown to act as an endogenous antioxidant and a free radical scavenger [96]. Indeed, ghrelin was shown to inhibit ROS formation and, on the other hand, to induce the production of antioxidant enzymes, thus supporting the redox balance [97]. This ability was reported in *in vitro* and *in vivo* models of several diseases [98-101] but never evaluated in models of mood disorders. For example, the systemic injection of ghrelin in a mouse model of Alzheimer’s Disease was associated with reduced ROS production and mitochondrial dysfunction in the hippocampus, contributing to the amelioration of cognitive impairment [101]. Furthermore, ghrelin has been reported to protect spinal cord motor neurons from apoptosis in cellular and animal models of Amyotrophic Lateral Sclerosis (ALS) [102-105]. Similarly, ghrelin was able to reduce apoptosis of dopaminergic neurons caused by the treatment with 1-methyl-4-phenyl-1,2,3,6-tetrahydropyridine (MPTP) in models of Parkinson’s Disease [106, 107].

Apart from ghrelin, synthetic molecules belonging to the family of growth hormone secretagogues (GHS) have also been shown to modulate microglia function and oxidative stress. Among them, the hexapeptides hexarelin and EP80317 showed protective effects toward different inflammatory stimuli [108-110], preserving neuronal activity, preventing apoptosis induced by hydrogen peroxide, and activating the peroxisome proliferator-activated receptor-γ (PPAR-γ) to mediate the internalization of low-density oxidized lipoproteins [111, 112].

## 5 NEUROINFLAMMATION AND OXIDATIVE STRESS: A THERAPEUTIC TARGET FOR MOOD DISORDERS?

Mood disorders, including MDD, have been associated with central and peripheral inflammation [113, 114]. Indeed, beyond the well-known increase of pro-inflammatory cytokines and chemokines in the blood of MDD patients, similar changes have also been found in the cerebrospinal fluid (CSF), and in postmortem brains. Accordingly, increased activated microglia were reported in postmortem brains of suicidal MDD patients [115].

Overall, these data suggested that dysregulated inflammatory processes in the brain might take part in MDD onset and development. Interestingly, preclinical models of depression also reported alterations of inflammatory markers, both peripherally and in the brain, strengthening the association between neuroinflammation and depressive-like behavior [116]. At the same time, both preclinical and clinical studies reported mitochondrial dysfunctions linked to increased oxidative stress in mood disorders [117, 118].

### 5.1 Neuroinflammation and Oxidative Stress as Targets of Antidepressants

Targeting neuroinflammation may represent a promising tool in the treatment of MDD. To date, clinical evidence has been mostly reporting the antiinflammatory effects of antidepressants on peripheral markers of inflammation. Indeed, the therapeutic effect of classical antidepressants was reported to be accompanied by a reduction of the peripheral levels of inflammatory markers in depressed patients [119]. Notably, ketamine, a glutamatergic drug with rapid-acting antidepressant properties, was also shown to rapidly modulate peripheral proinflammatory cytokines [120]. On the other hand, anti-inflammatory drugs such as non-steroid anti-inflammatory drugs or minocycline were reported to improve depressive symptoms in MDD patients [121, 122], corroborating the hypothesis that targeting inflammation may help to obtain an antidepressant effect.

Similarly, in stress-based animal models of depression, antidepressants were reported to rescue depressive-like behaviors together with decreasing the activation of microglial cells and the expression of pro-inflammatory cytokines in specific brain areas (especially the hippocampus, cortex and amygdala) (for a detailed review, see [123]). Moreover, ketamine rescued LPS-induced depressive-like behavior by reducing the expression of cytokines in the prefrontal cortex of rats [124] and inhibited microglia inflammation in LPS-treated BV2 cells *in vitro* [125].

Further, classical antidepressants have been shown to modulate the oxidative status in MDD patients, decreasing blood levels of critical enzymes in the production of free radicals such as malondialdehyde and superoxide dismutase while, on the other hand, increasing the levels of the antioxidant ascorbic acid [126, 127].

### 5.2 Putative Antidepressant Mechanisms of Ghrelin

Taken together, previous literature suggests that 1) ghrelin may exert antidepressant properties in both MDD patients and animal models of depression; 2) ghrelin can regulate neuroinflammation and oxidative stress in the brain, inducing neuroprotective effects in models of neurodegenerative diseases; 3) neuroinflammation and oxidative stress play a key role in the pathophysiology and progression of mood disorders; 4) the therapeutic effects of antidepressants are associated with reduced neuroinflammation and oxidative stress; 5) drugs with anti-inflammatory and antioxidant properties can exert antidepressant effects.

Thus, we may speculate that the behavioral antidepressant and anxiolytic effects of ghrelin might be dependent on its capability to regulate both neuroinflammation and oxidative stress, thus promoting neuroprotection and neuroplasticity. Indeed, disruptions in neuroplasticity pathways are considered a crucial factor in the pathophysiology of mood disorders, while stimulating molecular and cellular mechanisms of neuroplasticity may improve depressive symptoms. Importantly, GHS-R1a is highly expressed in the hippocampus and previous studies reported that elevated ghrelin levels induced by fasting enhance hippocampal neurogenesis and memory [128, 129]. Intriguingly, the stimulatory effect of ghrelin on adult neurogenesis seems to be mediated by increased levels of brain-derived neurotrophic factor (BDNF) [130].

**Fig. (1):**
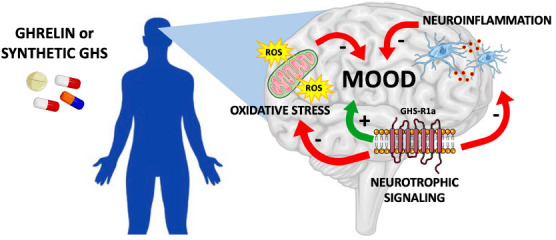
Putative pathways involved in the antidepressant and procognitive effects of ghrelin. Administration of ghrelin or its synthetic analogues may represent a promising therapeutical approach for mood disorders by dampening neuroinflammation and oxidative stress and promoting neurotrophic signaling downstream GHS-R1a activation. GHS: growth hormone secretagogue.

We thus speculate that antidepressant and procognitive effects of ghrelin might be mediated, on the one hand, by direct activation of neurotrophic intracellular signaling pathways downstream of GHS-R1a activation [109, 110, 131] and, on the other hand, indirectly through anti-inflammatory and anti-oxidative mechanisms (Fig. **1**). More studies are needed to understand how the modulation of the ghrelin system, which is intriguingly positioned at the interface between feeding circuitry, metabolism, and the HPA axis, can be exploited for the treatment of mood disorders. The potential role of ghrelin modulation in psychiatric diseases is further underscored by the notion that mood disorders are often associated with metabolic dysfunction and also show a high level of comorbidity with eating and metabolic disorders [86, 132, 133]. Thus, strategies targeting the ghrelin system could be of particular interest in depressed patients with appetite alterations or presenting comorbidity with eating or metabolic disorders. An interesting and never-tested hypothesis is that the combination of drugs targeting the ghrelin system with traditional antidepressants could potentially improve the therapeutic outcome in some patients and help in the personalization of treatment. Future research should explore the therapeutic potential of the modulation of the ghrelin system in psychiatric and metabolic disorders.

## CONCLUSION AND FUTURE PERSPECTIVES

Overall, the studies conducted to date demonstrate that the ghrelin system has the intriguing potential of orchestrating feeding, reward, and mood behaviors at the same time, thus potentially representing a novel therapeutic target for mood and eating disorders. Most of the reports concur in attributing psychotropic and procognitive functions to ghrelin, further confirming the relevance of the gut-brain axis in regulating mood and cognition. Nevertheless, the use of ghrelin, a peptide hormone, as a therapeutic drug would not be suitable due to the poor pharmacokinetics and limited oral activity of the molecule. However, in recent years, efforts have been made on the development of synthetic compounds to find selective and highly potent GHS-R1a ligands [134], and GHS-R1a agonists have attracted attention for their potential use in several pathologies [134-136]. This could represent an innovative therapeutic strategy to alleviate the suffering of patients with mood disorders and fight against treatment-resistant depression.

## Figures and Tables

**Table 1:** Changes of ghrelin and its receptor GHSR in stress models of depression.

**Model**	**Species**	**Strain**	**Sex**	**Effect**	**Tissue**	**mRNA/** **Protein**	**References**
Tail pinch Fasting	Mouse	ddY	M	↑ Ghrelin	Stomach	mRNA	[38]
Restraint, predator scent, FST, OF, noise stress, social stress, novel aversive environment	Mouse	C57BL/J6 and DBA	M	↑ Acyl-ghrelin	Plasma	Protein	[39]
CSDS	Mouse	C57BL/6J	M	↑ Acyl-ghrelin ↑ GHSR	Plasma HPC	Protein	[40]
Maternal separation	Mouse	CD1	M/F	↑ ghrelin	Plasma	Protein	[41]
Restraint stress	Mouse	ICR	M	↑ Des-acyl-ghrelin	Plasma	Protein	[42]
Water avoidance	Rat	Wistar Sprague-Dawley	F	↑ Octonoylated/des-octonoylated ghrelin	Plasma	Protein	[43]
Repeated restraint stress	Rat	Sprague-Dawley	M	↑ Acyl-ghrelin	Plasma	mRNA, Protein	[44]
Chronic Social Isolation	Rat	Wistar	M	↑ Acyl-ghrelin	Serum	Protein	[45]
Chronic Social Stress	Mouse	C57BL/6J	M	↑ Acyl-ghrelin ↑ GHSR	Plasma VMH	Protein	[46]
CUMS	Rat	Wistar	M	↑ Ghrelin	Serum	Protein	[47]
Postpartum maternal separation and immobilization stress	Mouse	ICR	F	↑ Ghrelin/acyl-ghrelin ↓ Ghrelin and GHSR ↓ GHSR	Serum HPC, PFC HPC, PFC	Protein mRNA Protein	[48]
CSDS	Mouse	C57BL/6J	M	↑ Acyl-ghrelin	Plasma	Protein	[49]
CUMS	Rat	Sprague-Dawley	M	↑ Ghrelin ↑ GHSR	PFC	mRNA	[50]
Social stress Social isolation	Rat	Wistar	M	= ghrelin ↑ ghrelin	Plasma	Protein	[51]
Immobilization stress	Rat	Sprague-Dawley	M/F	↑ Ghrelin	Serum	Protein	[52]
Social isolation	Mouse	C57BL/6	M/F	↑ Acyl-ghrelin (M) = Acyl-ghrelin (F)	Plasma	Protein	[53]
Immobilization stress	Rat	Long Evans	M	↑ Acyl-ghrelin	Plasma	Protein	[54]
CUMS	Rat	Sprague-Dawley	M	↓ Ghrelin	Serum	Protein	[55]
LPS	Rat	Sprague-Dawley	M	↓ Acyl-ghrelin ↓ Des-acyl-ghrelin	Plasma	Protein	[56]
Novelty	Mouse	C57BL/6J	M	↓ Acyl ghrelin	Plasma	Protein	[57]
CSDS	Mouse	CD1	M	↓ Ghrelin	Plasma	Protein	[58]
Early life social stress	Rat	n.d.	F	↑ GHSR	PVN	mRNA	[59]
PNS	Rat	Sprague-Dawley	M/F	↑ GHSR	vHPC PFC	mRNA	[60]
CUMS	Mouse	C57BL/6J	M	↑ Pre-proghrelin ↑ GHSR	HPC	mRNA mRNA, Protein	[61]
Foot shock	Rat	Long Evans	M	= Acyl-ghrelin	Plasma	Protein	[64]
Foot shock	Rat	Wistar	M	= ghrelin	Plasma	Protein	[65]
Water cage	Rat	Wistar	M	= ghrelin	Plasma	Protein	[66]
Water cage	Rat	Wistar	M	= ghrelin = acyl-ghrelin = des-acyl-ghrelin	Plasma	Protein	[67]

**Abbreviations:** CSDS: chronic social defeat stress, CUMS: chronic unpredictable mild stress, HPC: hippocampus, vHPC: ventral hippocampus, PFC: prefrontal cortex, PVN: paraventricular nucleus of the hypothalamus, VMH: ventral medial hypothalamus.

**Table 2:** Effects of exogenous administration of ghrelin, GHSR agonists and antagonists on mood in preclinical models.

**Model**	**Species**	**Strain**	**Sex**	**Treatment**	**Administration**	**Behavioural Effect**	**References**
Tail pinch Fasting	Mouse	ddY	M	Ghrelin	i.c.v.	Anxiogenic	[38]
CSDS	Mouse	C57BL/6J	M	Ghrelin	HPC micro-injection i.p. (10 days)	Antidepressant anxiolytic	[40]
CSDS	Mouse	C57BL/6J	M	Ghrelin	s.c.	Antidepressant anxiolytic	[49]
CUMS	Mouse Rat	C57BL/6J Sprague-Dawley	M M	Acyl-ghrelin ghrelin GHRP-6	i.p. i.c.v.	Antidepressant anxiolytic Antidepressant	[61]
Fasting	Rat	Sprague-Dawley	M/F	Acyl-ghrelin JMV2959	i.p.	Anxiolytic (F)	[62]
Olfactory Bulbectomy	Mouse	Albino Swiss	F	Ghrelin	i.c.v.	Antidepressant anxiolytic	[68]
Naive	Mouse	C57BL/6J	M	Ghrelin L-692,585 YIL781	dCA1 infusion	Impaired long-term memory acquisition	[69]
CSDS	Mouse	C57BL/6N	M	Acyl-ghrelin	s.c. (acute and osmotic pump)	No effect on social avoidance	[70]

**Abbreviations:** CSDS: chronic social defeat stress, CUMS: chronic unpredictable mild stress, s.c.: subcutaneous injection, i.c.v.: intracerebroventricular injection, i.p.: intraperitoneal injection.

**Table 3:** Transgenic models of ghrelin.

**Genotype**	**Species**	**Strain**	**Sex**	**Stress Paradigm**	**Ghrelin Level**	**Effect on Mood**	**References**
GHSR knockout	Mouse	C57BL/6J and DBA	M	Restraint, predator scent, FST, OF, social stress, noise stress, novel aversive environment	↑ Acyl-ghrelin	-	[39]
GHSR knockdown (AAV-shRNA)	Mouse	C57BL/6J	M	CSDS	-	↑ Depression- and anxiety-like behaviour	[40]
GHSR knockout	Mouse	C57BL/6J	M	CSDS	↑ Ghrelin	↑ Social avoidance	[49]
GHSR knockout	Mouse	C57BL/6J	M	CSDS	-	↑ Depressive-like behaviour	[71]
GHR knockout	Mouse	C57BL/6	M	Acute restraint stress	-	↑ Anxiety-like behaviour	[72]
GHSR knockout GHR knockout	Mouse	C57BL/6J	M	CRS	-	= Anxiety-like behavior ↑ Anxiety-like behaviour	[73]
GHSR knockout	Mouse	C57BL/6	M	CSDS	↑ Ghrelin	= Anxiety and despair-like behaviour	[74]
GHSR knockout	Mouse	C57BL/6J	M	-	-	= Fear acquisition, extinction and extinction retention ↓ Saccarine preference	[75]
GHSR knockout	Mouse	C57BL/6J	M	CSDS	↑ Acyl-ghrelin = des-acyl-ghrelin	↑ Social isolation	[76]

**Abbreviations:** CSDS: chronic social defeat stress, FST: forced swim, OF: open field.

## LIST OF ABBREVIATIONS

ACTH: Adrenocorticotropic Hormone
ALS: Amyotrophic Lateral Sclerosis
BDNF: Brain-derived Neurotrophic Factor
CCK: Cholecystokinin
CRF: Corticotropin-releasing Factor
CRH: Corticotropin-releasing Hormone
CSDS: Chronic Social Defeat Stress
CSF: Cerebrospinal Fluid
CUMS: Chronic Unpredictable Mild Stress
GHS-R1a: Growth Hormone Secretagogue Receptor 1a
GLP-1: Glucagon-like Peptide
GOAT: Ghrelin O-acyltransferase
HPA: Hypothalamic-pituitary-adrenal
HPC: Hippocampus
MDD: Major Depressive Disorder
MPTP: 1-methyl-4-phenyl-1,2,3,6-tetrahydropyridine
NLRP3: Nod-like Receptor Protein 3
PFC: Prefrontal Cortex
PPAR-γ: Peroxisome Proliferator-activated Receptor-γ
PTSD: Post-Traumatic Stress Disorder
PVN: Paraventricular Nucleus of Hypothalamus
PYY: Peptide YY
ROS: Reactive Oxygen Species
SSRIs: Selective Serotonin Reuptake Inhibitors
VMH: Ventral Medial Hypothalamus
